# Contextualized job crafting model: a quantitative study in the context of Pakistan

**DOI:** 10.3389/fpubh.2026.1839632

**Published:** 2026-06-09

**Authors:** Azra Aslam, Mahwish Kamran, Sohni Siddiqui

**Affiliations:** 1 Department of Educational Sciences, Iqra University, Karachi, Pakistan; 2 Institute for Educational Research, Bergische Universität Wuppertal, Wuppertal, Germany

**Keywords:** burnout, job crafting, job satisfaction, wellbeing, work environment, work life balance

## Abstract

**Introduction:**

One of the most widely studied topic in the educational research is teacher's wellbeing and rightly so, as resilient and well-supported teacher serves as an essential pillar for creating an effective and durable educational system. Researchers have long been engaged in critically reflecting on which dimensions must be encompassed within the conceptual framework of ‘wellbeing'. A great body of research has been done to study several aspects of wellbeing yet no simple combination of such factors has been identified which can readily constitute the most important factors, which shows that it is still a highly subjective area.

**Methods:**

In this research, which is a quantitative in nature, the influence of job crafting has been observed impacting on three dimensions of wellbeing; job satisfaction, work life balance and burnout, whereas work environment has been observed as the mediator. This study employed a quantitative cross-sectional research design. Data were collected through an adapted self-administered questionnaire from200 private high school teachers teaching international syllabi in schools across Karachi. Participants were selected through stratified sampling, while the schools were approached cluster-wise from the five major divisions of the city. All participating teachers had more than 10 years of teaching experience. The questionnaires were sent to them through emails after seeking permission from their respective school heads. The results have been analyzed through SPSS and AMOS.

**Results:**

Study results indicate that job crafting significantly improves teachers' work environment and job satisfaction while reducing burnout. Surprisingly, job crafting and a positive work environment were both found to have a significant negative impact on work-life balance, identifying a critical area for further research. Mediation analysis revealed that while the work environment does not significantly mediate job crafting's effect on burnout or work-life balance, it serves as a significant mediator for job satisfaction.

**Discussion:**

The study suggests that a supportive work environment strengthens the positive impact of job crafting on teachers' job satisfaction.

## 1 Introduction

The importance of the profession of teaching as the basis of all professions is imperative to leave no stone unturned in bringing in the best minds in the business, and to help them stay and thrive in the profession. Much research has been conducted on the aspects which help teachers prosper in their professions, however most of the studied factors are external

in nature such as school policies and workplace environment (1), type of leadership and workload (2) and many more. Even the personal and internal aspects of teachers which are studied in this context are more reactive in nature, such as burnout (3), stress management (4), and more. These factors are referred to as ‘reactive in nature' because they arise in an individual in response to specific negative external stimuli. However, in this research, the concept of job crafting is being studied which is fundamentally pro-active in nature (5). Job crafting is a rather new concept in the prospect of education (6), since it was initially studied in nursing and hospitality profession (7, 8). This term was coined by Wrzesniewski and Dutton (9), which means an individual's personal and proactive endeavor to tailor his job so as to match his own, unique skills, desires and tendencies, and as a result there is better output, increased sense of ownership, and reduced weariness and negativity (10).

**Figure 1:**
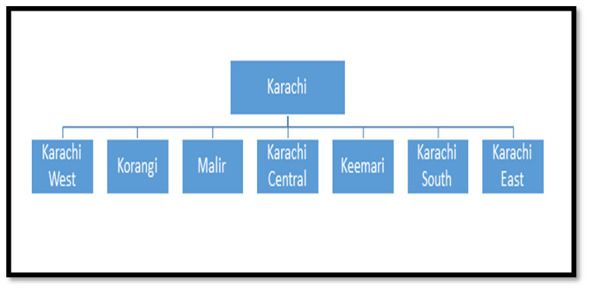
Divisions of Karachi.

This research is an attempt to study how does the practice of job crafting impacts the levels of work-life balance, burnout and job satisfaction among the high school teachers teaching international syllabi in different divisions of Karachi, Pakistan (for divisions refer to Figure 1). The purpose of this selection is that these institutions are reported to have pressing job demands such as rigorous curricula, exalted parental expectations, and performance pressure by the management, which create situations where experienced teachers are tend to devise job crafting practices so as to efficiently mitigate its negative impacts in the form of burnout thereby sustaining their wellbeing (10, 11). Besides that, there is a notable research gap, since the available research on job crafting is largely focused on primary schools or university level teachers (12, 13). A plausible explanation could be that access to private high schools for research purpose is restricted due to internal policies related to concern over reputation and ranking (14), secondly, compared to these high schools, universities are the places where research infrastructure is stronger, and that having access to funding, organizational support and ethic review boards is not difficult (15), and more importantly, in the context of Pakistan, limited attention has been given to the wellbeing of high school teachers, even though the country is committed to achieving Sustainable Development Goal 4 (SDG 4). Much of the national and international educational funding is directed toward primary education, resulting in comparatively less focus on the high school sector (16). In addition, previous studies have generally examined factors related to teacher wellbeing separately rather than exploring them together. Very few studies have considered the role of the work environment as a mediating factor, particularly in the context of private high schools. This gap in the literature indicates the need for further research in this area.

Despite increasing attention to teacher wellbeing, existing research remains fragmented in explaining the relationships among job crafting, job satisfaction, and burnout. Most studies have examined these variables separately, with limited focus on how teachers' proactive behaviors, such as job crafting, influence both positive outcomes (job satisfaction) and negative outcomes (burnout) within a unified framework (17). From the perspective of the Job Demands-Resources Model, employee wellbeing is shaped by the interaction of job demands and resources; however, job crafting as a proactive mechanism has not been sufficiently integrated into this process. Moreover, the mediating role of the work environment remains underexplored, particularly in developing contexts such as Pakistan. This study addresses these gaps by examining these relationships within a single conceptual model.

## 2 Literature review

### 2.1 Conceptual foundations of job crafting and the job demands-resource model (JD-R)

The phenomenon of job crafting refers to employees' proactive efforts to redesign and tailor their work-related tasks in ways that align with their personal strengths, interests, and preferences. It encompasses modifying task boundaries, reframing the perception of one's role, and shaping the quality of workplace relationships (18). Within the teaching profession, job crafting is particularly significant, as teachers constantly face multiple and often conflicting demands such as high workloads, large class sizes, administrative pressures, and emotional labor. Using the Job Demands-Resources (JD-R) Model as a framework, job crafting enables teachers to balance these demands with the resources available to them, ultimately helping them to sustain motivation, enhance satisfaction, and improve their wellbeing (10, 19).

For instance, teachers may increase structural resources by pursuing professional development (20), adopting digital learning tools (21), or adjusting instructional strategies to fit both student needs and their preferred pedagogical styles (22). They may also strengthen social resources through collaboration with colleagues, forming supportive peer networks, or engaging in mentoring relationships (23). On the other hand, job crafting allows teachers to reduce hindering demands by refining classroom management strategies, streamlining lesson preparation, or negotiating workload allocation with administrators (24). Moreover, teachers can also expand challenging demands by developing innovative projects, leading co-curricular activities, or experimenting with novel teaching approaches that increase the meaningfulness of their work (25).

These practices illustrate the JD-R Model's central proposition: when teachers proactively enhance job resources and regulate demands, they not only protect themselves from burnout but also foster higher levels of engagement and professional fulfillment (26). For example, designing project-based learning units can transform heavy curriculum expectations into stimulating challenges that energize both teachers and students. Likewise, cultivating positive teacher–student and teacher–parent relationships serve as a crucial social resource that reduces emotional strain while enhancing motivation (27). In this way, job crafting acts as a dynamic mechanism that enables teachers to actively align their work environment with the JD-R framework, maintaining a sustainable balance between demands and resources and promoting both effectiveness and wellbeing. The conceptual framework of this study is primarily grounded in the Job Demands-Resources Model, which provides a comprehensive explanation of how job demands and job resources jointly shape employee wellbeing outcomes such as job satisfaction, burnout, and work–life balance. Within this framework, job crafting is understood as a proactive behavioral strategy that enables employees to modify their job demands and resources to achieve better psychological and professional outcomes.

Furthermore, the assumptions of the Job Demands-Resources Model are also supported by perspectives from Self-Determination Theory, which emphasizes the importance of autonomy, competence, and relatedness in enhancing motivation and psychological wellbeing (28). According to Self-Determination Theory (SDT), environments that satisfy the basic psychological needs for autonomy, competence and relatedness foster autonomous motivation, which drives proactive employee behavior (28). Autonomy is defined as the desire to act with volition and personal choice; competence is defined as the desire to seek mastery over tasks and develop one's abilities; and relatedness is defined as the desire to feel connected to a community and supported by others (29). As these three needs are fundamental to optimal human functioning, their fulfillment in the workplace is essential for psychosocial adjustment and employee wellbeing (30). Furthermore, SDT provides a robust theoretical basis for explaining how job crafting contributes to wellness by detailing the mechanism through which individuals proactively adapt their roles to better satisfy these core psychological requirements. In educational settings, teachers who are able to shape their work according to their strengths and professional needs are more likely to experience satisfaction, engagement, and overall wellbeing.

Another theoretical approach to explaining job crafting has been taken from the work engagement theory. In line with the model of engagement developed by Schaufeli and Bakker (2004), work engagement is defined as a sustained mental and emotional state comprising three dimensions: vigor, dedication and absorption (31). Within this framework, the relationship between job crafting and engagement has become a central focus of organizational research, with studies consistently demonstrating a strong positive correlation (32). Specifically, proactive behaviors such as increasing structural and social job resources or seeking out new challenges serve as significant predictors of heightened engagement levels (33). This is further supported by Rudolph et al. (2017) meta-analysis of 122 independent samples concluded that job crafting is fundamentally linked to increased work engagement and other positive contextual variables (34). This reinforces the theory that employees who actively shape their roles experience a more profound sense of connection and energy in their work.

Together, these theoretical perspectives justify the inclusion of job crafting, work environment, job satisfaction, and burnout in a single integrated model, as they collectively explain both motivational and strain-based processes influencing teacher wellbeing.

### 2.2 Job crafting

Job crafting is increasingly recognized as a self-initiated approach through which employees reshape their jobs to better align with their talents, skills, and interests (35). Wrzesniewski and Dutton (2001) identified three core forms of job crafting: task crafting, where employees modify the nature or scope of their tasks; relational crafting, which involves redefining social interactions at work; and cognitive crafting, where employees reframe the way, they perceive their work (9). These forms of crafting enable employees to bend the formal boundaries of their jobs, ultimately fostering a stronger sense of ownership and engagement.

### 2.3 Work environment as a mediator

The work environment plays a critical mediating role in the relationship between job crafting and teacher wellbeing (36). Mushtaq and Mehmood argue that supportive and positive environments empower teachers to actively engage in crafting behaviors (37). Such environments are characterized by resources like peer support, autonomy, teaching materials, and openness to innovation, collectively referred to as job resources (6). Teachers in these settings report higher confidence in crafting their jobs, which enhances their wellbeing (38).

### 2.4 Job crafting and teacher wellbeing

Wellbeing broadly reflects an individual's evaluation of their quality of life, encompassing both the presence of positive emotions (e.g., satisfaction, happiness) and the absence of negative states such as burnout (39). Teachers who enjoy autonomy in aligning tasks with personal preferences, perceive their work as purposeful, and balance professional with personal responsibilities tend to report stronger wellbeing (40–42). Evidence from the Chinese educational context further confirms that job crafting is associated with higher workplace wellbeing among teachers (43).

### 2.5 Job crafting and burnout

Job crafting also mitigates burnout, a condition defined by emotional exhaustion, depersonalization, and reduced personal accomplishment (44, 45). Studies show that especially cognitive and relational crafting alleviate emotional fatigue by enhancing job satisfaction and personal fulfillment (11, 18, 36). Since burnout erodes wellbeing through fatigue, loss of self-worth, and cynicism (3), crafting serves as a protective mechanism for teachers against prolonged occupational stress.

### 2.6 Job crafting and work–life balance

Work–life balance represents the harmony individuals achieve between professional responsibilities and personal obligations (46). For teachers, this balance is critical in sustaining both physical and mental wellbeing, family life, job satisfaction, and organizational commitment (17, 47–50). In the post-COVID context, where boundaries between work and personal time are increasingly blurred, job crafting strategies such as task reorganization, relationship management, and cognitive reframing help teachers maintain equilibrium (42, 51).

### 2.7 Job crafting and job satisfaction

A strong positive relationship also exists between job crafting and job satisfaction. By proactively modifying their tasks and social relationships, employees feel psychologically empowered, which enhances their satisfaction at work (52). Job satisfaction itself is the positive emotional response individuals develop toward their work experiences (53). Teachers with higher job satisfaction report greater wellbeing, as satisfaction negatively correlates with burnout and positively correlates with life satisfaction (54).

Recent studies conducted between 2023 and 2025 further strengthen the understanding of job crafting in relation to teachers' wellbeing, particularly within private and international school contexts. Contemporary research continues to position job crafting as a proactive strategy that enhances teachers' psychological and professional outcomes by enabling them to manage job demands and utilize available resources effectively.

A recent study by Aslam et al. in the context of private schools in Karachi found that job crafting significantly improves teachers' job satisfaction, reduces burnout, and enhances overall wellbeing when supported by a positive work environment (55). The study highlights that teacher actively engage in task and relational adjustments to cope with heavy workloads and institutional demands, which aligns strongly with the principles of the Job Demands-Resources Model.

Similarly, recent international evidence suggests that job crafting contributes to reducing teacher burnout by strengthening job resources and increasing perceived autonomy. Large-scale educational studies further indicate that leadership support and workplace resources play a critical role in shaping teacher wellbeing outcomes, reinforcing the importance of organizational context (17).

Further, recent evidence highlights that job crafting enhances job satisfaction indirectly through supportive organizational climates, where autonomy, collegiality, and institutional encouragement enable teachers to reshape their work roles more effectively (56).

Although job crafting is a relatively new concept (6), research in Pakistan shows promising trends. Studies highlight that teacher wellbeing is positively influenced by job crafting, particularly in supportive work environments where autonomy and resources enable teachers to adapt their roles effectively (12, 13). The current research examines the role of job crafting among educators in Pakistan's metropolitan international schools. By focusing on these high-pressure environments, the study aims to understand how teachers navigate the significant stressors and intensive curriculum demands inherent in international education.

### 2.8 Defining related key concepts

To fully understand the interplay between job crafting and teacher wellbeing, it is essential to clarify related constructs:

- *Wellbeing:* A holistic, subjective state that extends beyond the absence of illness, reflecting positivity and hopefulness in relation to physical, social, and economic conditions (57).
- *Work–life Balance:* Employees' perceptions of balancing professional and personal/familial roles, with direct implications for health, satisfaction, and organizational commitment (46, 50).
- *Job Satisfaction:* The positive feelings arising from autonomy, growth opportunities, and supportive organizational culture (58, 59).
- *Burnout:* A professional phenomenon recognized by the WHO, characterized by exhaustion, cynicism, and a diminished sense of accomplishment (44).
- *Work Environment:* The physical, social, and organizational context in which employees perform tasks, encompassing conditions that directly affect performance and satisfaction (36).

## 3 Research methodology

### 3.1 Research design

The purpose of this study is to assess the impact of job crafting on teachers' work-life balance, burnout and job satisfaction, with the school environment acting as the mediator. This quantitative cross-sectional research study uses self-administered questionnaires to collect data. A quantitative design was considered appropriate because the study aimed to examine the relationships among measurable variables through numerical data analysis. The questionnaire was adapted under the supervision of an expert to ensure reliability and validity. To ensure ethical considerations were upheld, a consent letter and the survey form were first sent to school heads, and only those schools that gave consent were approached. This study has cross-sectional approach since it provides a snapshot of teachers' behavior at a specific time without needing any long term follow up. The cross-sectional design was selected because it enabled the researcher to examine teachers' perceptions, experiences, and workplace behaviors within a defined timeframe in a practical and time-efficient manner. Since it was a cross-sectional study, there was a specific time period for data collection, spanning from September 2023 to September 2024. To analyse the results and test the hypothesis AMOS and SPSS 22 were used.

### 3.2 Research questions

This research has studied the following research questions.

RQ 1) Is there an impact of job crafting on work-life balance, work environment and burnout of teacher?

RQ 2) Is there an impact of work environment on job satisfaction, burnout and work life balance of teachers?

RQ3) What is the mediating impact of work environment on the relationship between job crafting and work-life balance of teachers; job satisfaction and burnout?

### 3.3 Research instrument

The research instrument consists of 30 questions other than demographics to measure five variables for the study. The initial part collects the demographic information of the respondents; age, gender, experience, and number of trainings received. However, the later part is collectively adapted using Likert scale by combining five questionnaires to assess teachers' responses for each of the five variables. Statements were measured using a 5-point Likert scale, where:

SD = Strongly Disagree (1)
D = Disagree (2)
N = Neutral (3)
A = Agree (4)
SA = Strongly Agree (5)

The instrument consists of the following variables:

- *Job Crafting:* Job crafting is assessed by the questions adapted by the questionnaire by Slemp and Vella (60). It's a 19-item based questionnaire which can effectively assess all three dimensions of job crafting—task, relational and cognitive crafting. We selected a specific subset of 7 items from the JCQ that exclusively focused on the employees' actions and behaviors related to modifying the tasks they perform. Its Cronbach alpha is 0.9, with 4 items, and, it has constantly demonstrated high validity and reliability (61). Example item: “I arrange my work tasks to fit my personal strengths.”
- *Work Environment:* Work environment was assessed by taking 6 items from SLEQ, the scale of Fisher and Fraser (62), which is highly relevant in current studies as well and shows strong validity and reliability with Cronbach's alpha = 0.86 (63). Example item: “Teachers are encouraged to be innovative in this school.”
- *Work Life Balance:* The dependent variables, work-life balance is assessed by the 6 items out of 19-item scale created by Fisher Mc-Auley et al. (64), which displays strong internal consistency and reliability (Cronbach's alpha = 0.93) despite having undergone multiple adaptations in varied contexts (65). Example item: “My job makes it difficult to maintain the kind of personal life I would like.”
- *Burnout:* Burnout was assessed by Maslach and Jackson's (44) 22-item scale. However, in this study five items of burnout were used. Its Cronbach's alpha values ranging from 0.82, showing high internal consistency and reliability. Example item: “I feel burned out because of my teaching work.”
- *Job Satisfaction:* Job satisfaction, was measured by the 6 items from the scale devised by Pepe et al. (66) whose reliability is ensured by Item Response Theory Analysis. Example item: “I am satisfied with the organization of work in my school.”

### 3.4 Sampling technique

The teachers working in the private high schools of Karachi are the main focus of the study, who are in the profession for the past 10 years, working in the schools which are teaching international syllabi, and are in business for more than 15 years, as well as catering to all genders of the students. Given the unknown population size, Cochran's large-population formula was applied (*p* = 0.05; 95% confidence). Initially, the sample size was 208, however after removing the outliers the final number of respondents remained 200. This taken sample size of 200 represents implied margin of error ~± 6.9%, meeting the precision target for key proportion estimates (67). We used a stratified random sampling technique. The city of Karachi was divided into seven districts, and a random sample was chosen from each district. Finally, in the spirit of inclusivity and uniformity, a thorough systematic online-search was done to identify the schools in each of the seven districts of Karachi, and then selected.

### 3.5 Data collection

Data were collected through an online questionnaire. Preliminary screening of the dataset was conducted using SPSS to ensure accuracy and suitability for further analysis. The screening process revealed no missing values; however, eight cases were identified as outliers.

### 3.6 Respondent profile

For this study 200 valid cases were used, 47 (23.5%) were male, and 153 (76.5%) were female teachers. Similarly, 67 respondents (33.5%) were aged between 40–45 years, 75 (32.5%) aged between 45–49 years, 41 (20.5%) aged between 50–54 years, and 27 (13.5%) were of 55 years, and above. Furthermore, 40 (20.0%) teachers had 10 years' experience, 71 (35.5%) had 11–15 years, and 89 (44.5%) had 15 years+ experience. As for the number of trainings, 8 teachers (4.0%) reported no trainings, 38 (19.0%) reported few trainings, and 154 (77.0%) shared to have taken multiple trainings (Refer to Table 1).

**Table 1:** Respondents' profile.

Demographics	Indicators	Frequency	Percentage
Gender	Male	47	23.5
	Female	153	76.5
Age	40–44	67	33.5
	45–49	65	32.5
	50–54	41	20.5
	55+	27	13.5
Experience	10 years	40	20.0
	15 years	71	35.5
	15+ years	89	44.5
No. of trainings	None	8	4.0
	Few	38	19.0
	Multiple	154	77.0

### 3.7 Ethical considerations

All participants were sent a consent form. They were clearly informed that they could withdraw at any time. The purpose of the study and the process were also explained to them. Only participants who had given their consent were included in the study. Strict confidentiality and ethical standards were maintained throughout the research process to ensure that no personal or institutional identities could be revealed. The researcher completed the ethical review form and submitted it to Iqra University's research unit. Departmental approval was received on 1 August 2023, prior to proceeding with the fieldwork related to the research.

## 4 Analysis of results

### 4.1 Factor analysis of the instruments

**Figure 2:**
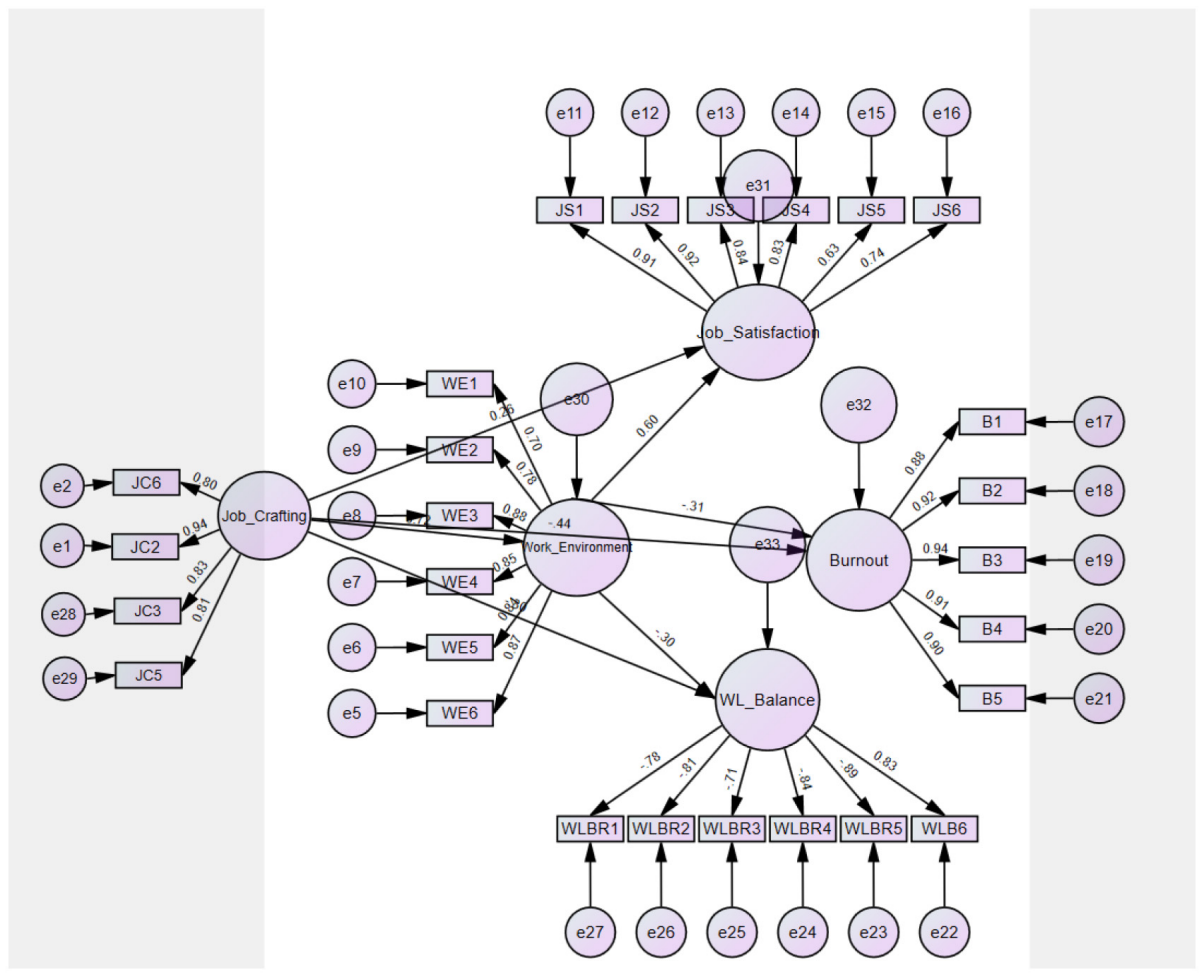
Impact of job crafting on work environment, work-life balance, burnout and job satisfaction.

For this study, confirmatory factor analysis was performed using AMOS (refer to Table 2 and Figure 2).

**Table 2:** Factor loading and construct validity.

Construct	Items	Factor loadings	Cronbach's alpha	Mc Donald's omega	Average variance extracted
Job crafting (JC)	JC1	0.94	0.912	0.912	78%
	JC2	0.83			
	JC3	0.81			
	JC4	0.80			
Work environment (WE)	WE1	0.70	0.925	0.926	
	WE2	0.78			
	WE3	0.88			
	WE4	0.85			
	WE5	0.84			
	WE6	0.87			
Job satisfaction (JS)	JS1	0.91	0.920	0.923	
	JS2	0.92			
	JS3	0.84			
	JS4	0.83			
	JS5	0.63			
	JS6	0.74			
Work life balance (WLB)	WLB1	0.78	0.920	0.919	
	WLB2	0.81			
	WLB3	0.71			
	WLB4	0.84			
	WLB5	0.89			
	WLB6	0.83			
Burnout (B)	B1	0.911	0.960	0.961	
	B2	0.938			
	B3	0.947			
	B4	0.933			
	B5	0.919			

JC, job crafting; WE, work environment; JS, job satisfaction; WLB, work-life balance; B, burnout.

### 4.2 Reliability, convergent validity, and model fitness

A common way of assessing the reliability of the tool is Cronbach's Alpha and McDonald's Omega with the typical threshold value set as 0.7 or higher (68, 69). Another important aspect of an instrument's legitimacy is its construct validity which is measured by Average Variance Extracted (AVE) whose recommended threshold is 50% or higher and factor loading values being above 0.6. These measures identify that reliability and convergent validity criteria is met (68) (refer Table 2). SPSS software is used for measuring reliability and validity of the instrument.

Model fit indices were calculated using AMOS. The results yielded a PCFI of 0.709 and a PNFI of 0.674; both values exceed the 0.50 threshold recommended by Schermelleh-Engel et al. (70).

### 4.3 Hypothesis testing (direct effects)

To test the hypothesized model, regression weights were calculated using AMOS software to establish the relationships between the variables (see Table 3). The results indicate a significant positive impact of job crafting on the work environment and job satisfaction, and a negative impact on burnout. This clearly shows that the use of job crafting techniques has a positive influence on the work environment and job satisfaction, and decreases burnout among teachers. However, the results concerning the impact of job crafting on work-life balance are surprising, as it has a significant but negative impact. Similarly, the work environment positively and significantly influences job satisfaction and negatively influences burnout, showing that a better work environment results in greater satisfaction among teachers and decreases their level of burnout. However, the negative correlation between work environment and work-life balance is surprising and requires further investigation. The discussion section highlights the possible reasons for these negative correlations, as well as their practical implications.

**Table 3:** Hypothesis testing.

Hypothesis	Path	Estimates	S. E	C.R.	*p*-value	Decision
There is strong and significant correlation between job crafting work environment among teachers.	JC → WE	0.622	0.056	11.206	^***^	Accepted (positive effect)
There is strong and significant correlation between job crafting and burnout among teachers.	JC → B	−0.375	0.075	−4.962	^***^	Accepted (negative effect)
There is strong and significant correlation between job crafting and job satisfaction of teachers.	JC → JS	0.234	0.068	3.422	^***^	Accepted (positive effect)
There is strong, significant correlation between job crafting and work-life balance of teachers.	JC → WLB	−0.298	0.102	−2.913	0.004	Accepted (negative effect)
There is strong and significant correlation between work environment and burnout of teachers.	WE → B	−0.308	0.087	−3.544	^***^	Accepted (negative effect)
There is strong and significant correlation between work environment and job satisfaction of teachers.	WE → JS	0.636	0.085	7.511	^***^	Accepted (negative effect)
There is strong, significant correlation between work environment and work-life balance of teachers.	WE → WLB	−0.349	0.119	−2.928	0.003	Accepted (negative effect)

^***^ = significant at level of 0.01.

### 4.4 Mediation analysis

To examine the mediating role of the work environment, a mediation analysis was conducted using the bootstrapping method (5,000 resamples) to derive two-sided bias-corrected confidence intervals (BCCI). Results indicated that the standardized indirect effects of job crafting on both work-life balance (β = −0.216, *p* = 0.107) and burnout (β = −0.225, *p* = 0.074) were not statistically significant at the 0.05 level, suggesting that the work environment does not serve as a mediating mechanism for these specific outcomes. However, a significant indirect effect was observed for job satisfaction (β = 0.437, *p* = 0.024). Specifically, for every one-standard-deviation increase in job crafting, job satisfaction increases by 0.437 standard deviations through the mediating influence of the work environment, independent of any direct effects (refer to Table 4).

**Table 4:** Mediation analysis.

Path	Direct effect (β)	Indirect effect (β)	Total effect (β)	95% CI (lower, upper)	*P*-value (indirect)	Result
Job crafting➔ work environment ➔work life balance	*−0.297*	*−0.216*	−0.513	−0.465, 0.067	*0.107*	*Insignificant*
Job crafting➔ work environment ➔job satisfaction	0.259	*0.437*	0.696	0.183, 0.708	*0.024*	*Significant*
Job crafting➔ work environment ➔burnout	*−0.439*	*−0.225*	−0.664	−0.499, 0.032	*0.074*	*Insignificant*

## 5 Findings and discussion

The purpose of this study was to examine the impact of job crafting on teachers' job satisfaction, burnout, and work–life balance, with work environment as a mediating factor in relation to the JD-R Model. The study focused on teachers with more than 10 years of teaching experience in schools offering international syllabi and operating for more than 15 years. By exploring these dimensions of teachers' wellbeing, the study highlights how job crafting interacts with workplace resources to sustain teachers' professional and personal functioning.

Consistent with earlier research, the findings reveal that teachers who engaged in job crafting practices reported higher levels of job satisfaction (52, 55). This relationship was further strengthened by the presence of a supportive work environment. Mediation analysis further revealed that the work environment did not significantly mediate the relationship between job crafting and work–life balance or burnout, although direct and significant impact of both job crafting and work environment are observed on work life balance and burnout. This indicates that while a supportive work environment contributes to lower burnout, it does not explain the mechanism through which job crafting influences burnout in this study. However, a significant indirect effect was found for job satisfaction, indicating that job crafting enhances job satisfaction through the work environment. This suggests that a supportive work environment strengthens the positive impact of job crafting on teachers' job satisfaction. Previous studies confirm that when teachers perceive their organizational environment as collegial and resourceful, job crafting is more effective in fostering satisfaction and reducing stress (6, 61). From the perspective of the Job Demands-Resources Model, this indicates that job resources (such as supportive environments) enhance motivational processes, leading to higher job satisfaction among teachers.

With respect to burnout, teachers who engaged in job crafting reported significantly lower levels of burnout, indicating reduced exhaustion and disengagement. This supports recent evidence that job crafting helps buffer job demands and sustain motivation within supportive organizational contexts (18). From the perspective of the Job Demands-Resources Model, this finding can be explained by the idea that job crafting enhances job resources and helps teachers manage excessive demands, thereby reducing strain and emotional exhaustion.

In contrast, findings for work–life balance were surprising. The results demonstrated a negative influence of job crafting and work environment on the work-life balance, differing from prior studies (56). This suggests that contextual and cultural factors may shape these relationships. From the perspective of the JD-R Model and Work Engagement Theory, increased job resources and engagement may intensify work involvement, leading to work-to-home spillover and reduced recovery time. Within the framework of the Job Demands-Resources Model, this may indicate that although job crafting increases motivational and engagement-related resources, it may simultaneously intensify work involvement, leading to an imbalance between job demands and personal recovery time. From the perspective of the Job Demands–Resources Model (36) and Work Engagement Theory (71), increased job resources such as job crafting and a supportive work environment enhance engagement, leading teachers to become more immersed in their roles. However, this heightened involvement may blur work and personal boundaries, causing work-to-home spillover and a perceived decline in work–life balance, despite improvements in job satisfaction and reduced burnout. Job crafting, or changing tasks or relationships at work, requires extra cognitive and emotional effort. In a South Asian context, where resources are scarce (72), job crafting often involves taking on extra informal responsibilities to improve classroom functioning. While this makes teachers feel more satisfied and less burnt out because they feel in control, the time and energy required to maintain these changes can leave them exhausted and have a negative impact on their family life. One other possible reason can be that the work-life balance instrument has not captured the true effects, and improvements could be made by using a different instrument.

In the Pakistani context, female teachers often bear disproportionate family and caregiving responsibilities alongside professional duties (73), which may restrict the potential benefits of job crafting for achieving balance. However, it is important to note that the study sample also included male teachers, who reported similar constraints related to workload intensity, rigid schedules, and modest economic returns in the teaching profession. Gender differences were not explicitly tested, but future studies should investigate whether men and women experience job crafting differently in terms of work–life balance outcomes (11). A possible explanation for the relatively weaker effect but significant negative effect on work–life balance of work environment and job crafting is the influence of unmeasured factors such as cultural expectations, organizational policies, and limited institutional resources. Previous literature also highlights that structural barrier, particularly in South Asian contexts, often limit the extent to which individual strategies like job crafting can compensate for systemic shortcomings (6). Consistently, mediation analysis showed that the work environment did not significantly mediate this relationship, indicating that work–life balance may be shaped more by broader contextual factors.

## 6 Conclusion

The results of this study show that job crafting significantly impacts teachers' job satisfaction and negatively affects their burnout. However, the effect of job crafting on work-life balance was observed to be negative, which is inconsistent with the literature. This peculiar outcome may be explained by the emblematic social and cultural fabric of Pakistani society, where rigid organizational policies, modest pay scales for teachers and gender-specific roles for working women also play a key role. Therefore, to strike a balance between personal and practical lives, fundamental and systematic changes are needed at policy and cultural levels.

## 7 Theoretical and practical implications

The findings underscore that schools should not only encourage teachers to proactively craft their jobs but also create supportive organizational environments that make such practices more effective. Professional development programs can integrate job crafting training, while school leaders should implement flexible policies and allocate adequate resources to reduce stressors. Addressing gendered expectations, such as providing family-friendly policies and flexible scheduling, can further enhance teachers' ability to balance work and personal responsibilities.

Furthermore, it is important to recognize that, although job crafting can increase job satisfaction, this should not be at the expense of work-life balance. The current study highlights a significant risk: job crafting may actually have a negative impact on this balance. If this imbalance is not addressed, it can lead to long-term strain on personal relationships. Consequently, organizations should proactively implement internal communication and training programmes emphasizing that employee wellbeing is as vital to institutional success as professional performance.

In sum, the study demonstrates that job crafting significantly enhances teachers' job satisfaction and reduces burnout. By applying the JD-R framework in the Pakistani educational context, the study offers evidence that proactive individual strategies, combined with organizational support, are essential for sustaining teacher wellbeing. Future research should incorporate gender analyses and examine contextual moderators to develop more tailored interventions that foster sustainable teaching careers.

## 8 Limitations

As for the limitations, this study only reflects the experience of teachers working in private high schools in Karachi which teach international syllabi, and are operating in areas in Karachi, only. This study is delimited to teachers working in private high schools in Karachi that offer international syllabi and are located within selected areas of the city. Furthermore, the perspective of only those teachers has been recorded who have been in business 10 years or more and only teachers with a minimum of 10 years of teaching experience were included in the study. The sample included a higher proportion of female respondents (approximately 76%), which reflects the actual gender composition of teachers in the selected private international schools in Karachi. However, this gender imbalance may limit the generalizability of the findings across more gender-balanced populations. Therefore, future studies should aim to include more balanced gender representation to further validate and generalize the results across diverse educational contexts. In addition to that, wellbeing is a broad umbrella term, yet only three dimensions have been focused, therefore, future research has a great capacity to include diverse types of schools, teachers with varied demographics, and other aspects of wellbeing, so as to achieve a wider understanding in addressing the gaps, and devising effective strategies to enhance teachers' wellbeing in diverse contexts.

## 9 Future research direction

Future research may include teachers from public schools and other educational contexts to improve generalizability. Studies may also explore additional dimensions of teacher wellbeing such as psychological resilience, emotional exhaustion patterns, and organizational commitment. Longitudinal and mixed-method designs are recommended to better understand how job crafting evolves over time. Future studies should also examine gender differences and include contextual moderators such as leadership style, organizational culture, and policy environment to better explain variations in work–life balance outcomes.

## Data Availability

The original contributions presented in the study are included in the article/supplementary material, further inquiries can be directed to the corresponding author.
